# Glycemic Index and Pregnancy: A Systematic Literature Review

**DOI:** 10.1155/2010/282464

**Published:** 2011-01-02

**Authors:** Jimmy Chun Yu Louie, Jennie C. Brand-Miller, Tania P. Markovic, Glynis P. Ross, Robert G. Moses

**Affiliations:** ^1^Discipline of Nutrition and Metabolism, School of Molecular Bioscience, The University of Sydney, Sydney, NSW 2006, Australia; ^2^Department of Endocrinology, Royal Prince Alfred Hospital, Camperdown, NSW 2050, Australia; ^3^Illawarra Diabetes Service, South East Sydney & Illawarra Area Health Service, Wollongong, NSW 2500, Australia

## Abstract

*Background/Aim*. Dietary glycemic index (GI) has received considerable research interest over the past 25 years although its application to pregnancy outcomes is more recent. This paper critically evaluates the current evidence regarding the effect of dietary GI on maternal and fetal nutrition. *Methods*. A systematic literature search using MEDLINE, EMBASE, CINAHL, Cochrane Library, SCOPUS, and ISI Web of Science, from 1980 through September 2010, was conducted. *Results*. Eight studies were included in the systematic review. Two interventional studies suggest that a low-GI diet can reduce the risk of large-for-gestational-age (LGA) infants in healthy pregnancies, but one epidemiological study reported an increase in small-for-gestational-age (SGA) infants. Evidence in pregnancies complicated by gestational diabetes mellitus (GDM), though limited (*n* = 3), consistently supports the advantages of a low-GI diet. *Conclusion*. There is insufficient evidence to recommend a low-GI diet during normal pregnancy. In pregnancy complicated by GDM, a low-GI diet may reduce the need for insulin without adverse effects on pregnancy outcomes. Until larger-scale intervention trials are completed, a low-GI diet should not replace the current recommended pregnancy diets from government and health agencies. Further research regarding the optimal time to start a low-GI diet for maximum protection against adverse pregnancy outcomes is warranted.

## 1. Introduction

Recent data suggest that mean birth weight has increased over time in many developed nations [1, 2]. Birth weight shows a “U” shaped relationship with adult obesity, such that both small and large babies are at increased risk [3, 4]. The long-term effects of higher body fat at birth are now attracting attention [5, 6]. Increasing birth weight was independently and linearly associated with increasing prevalence of obesity at age of 7 years in the Avon cohort of children [7]. In particular, infants defined as large for gestational age (LGA; birth weight ≥ 90th percentile) at birth remained in the upper tertile of weight throughout early childhood [8], even after controlling for social status, birth order, and maternal weight. Of concern, excessive fetal growth confers increased risk for obesity and diabetes that carries over to successive generations [9–11]. Maternal hyperglycemia leading to fetal hyperinsulinemia has been suggested to be responsible for some of this increase in risk [12]. These findings imply that avoidance of LGA or high body fat at birth should be a target for population-based obesity prevention strategies*.*

Since elevated maternal blood glucose levels are well recognized to contribute to excessive fetal growth [13], strategies to lower maternal postprandial blood glucose levels such as a low glycemic index (GI) diet may improve pregnancy outcomes. The potential mechanisms of the benefits of a low-GI diet may be due to the reduction in the rise of the postprandial blood glucose level, which in turn reduces hyperinsulinemia [14] and oxidative stress [15]. A low-GI diet that reduces postprandial glucose spikes may therefore represent a logical and healthy way of eating during pregnancy benefiting the future health of the offspring.

This paper aimed to systematically examine the current evidence linking diets with either a high or low GI to maternal nutrition and pregnancy outcomes.

## 2. Methodology

### 2.1. Search Strategies

**Figure 1:**
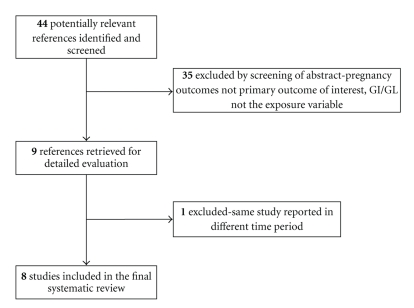
Papers identified through study selection process.

A literature search using MEDLINE, EMBASE, CINAHL, Cochrane Library, SCOPUS, and ISI Web of Science, from 1980 through September 2010, was conducted with the Medical Subject Headings (MeSH) “Glycemic Index,” “Pregnancy Outcomes,” “Diet,” and other relevant terms (see the appendix for complete search strategy). The search was restricted to human studies with no restrictions on age or ethnicity. Only articles published in English were included, and a manual search of references cited by the identified studies was also undertaken. To determine the eligibility of the identified studies, the abstracts of the 44 identified studies were screened, and the full text of the article was reviewed when the abstract did not provide enough information. Studies that included GI and/or GL as the exposure variable and pregnancy outcomes as the primary outcome variable were included. The flow of study analysis is shown in Figure 1. A total of nine studies were included in this systematic review. Due to the small number of studies found and the high heterogeneity of the study populations and outcomes, a meta-analysis could not be performed.

## 3. Results

### 3.1. Evidence in Normal Pregnancy

Table 1 shows the characteristics of the five studies (two epidemiological and three interventional) examining the association between GI/GL and pregnancy outcomes in healthy pregnancies. 

#### 3.1.1. Epidemiological Studies

In the Camden Study [16], the investigators assessed the diets of 1,082 healthy pregnant women using 24-hour recalls. They found that HbA_1c_ and plasma glucose increased by 0.006% and 0.013 mmol/L (both *P* < .05) per unit increase in the dietary GI, respectively. In addition, infants of women who had a dietary GI < 50 had a significantly lower birth weight (116 g lower, standard error = 34 g; *P* < .05). However, compared to those with a dietary GI of 54–56, those with a dietary GI less than 50 had a 75% (95% CI: 10–177%) increased risk of giving birth to small-for-gestational-age (SGA; birth weight ≤ 10th percentile) infants, with no link between high dietary GI and risk of LGA infants. 

The study by Deierlein et al. [17], which assessed the dietary GL of the subjects by a 110-item semiquantitative FFQ, found no relationship between dietary GL at 26–29 weeks gestation and total gestational weight gain and weight gain ratio.

#### 3.1.2. Intervention Studies

All three identified intervention trials support the hypothesis that low-GI diets may safely reduce the risk of macrosomia. The study by Clapp [18] was the first to investigate the effect of a low-GI diet on the pregnancy outcome of healthy gravidas. The 12 participants in this study first followed a low-GI weight maintenance diet from before pregnancy until eight weeks gestation and were then randomized to either continue the low-GI diet (“aboriginal” carbohydrate diet) or to an isoenergetic high GI (“cafeteria” carbohydrate) diet. He found that mothers on a high-GI diet gained more weight (mean ± SE: low GI 11.8 ± 2.3 kg versus high GI 19.7 ± 1.2 kg; *P* < .01). Infants whose mothers were on the high-GI diet had higher birth weight (mean ± SE: low GI 3.27 ± 0.12 kg versus high GI 4.25 ± 0.11 kg; *P* < .01), and higher fat mass (mean ± SE: low GI 301 ± 50 g versus High GI 402 ± 80 g; *P* < .01). 

In the study by Moses et al. [19], 70 healthy women with singleton pregnancy in weeks 12–16 of gestation were recruited and allocated to either a low-GI or a conventional diet, both matched for macronutrients, and 62 women completed the study. They found that women who consumed the low-GI diet had a decreased prevalence of LGA infants (3% versus 33% in the conventional diet group) while the prevalence of SGA was not significantly different (9% versus 7%). In the 2-year followup of the original study [20], they found that subjects had reverted to their baseline diet despite the intensive dietary advice given during pregnancy, while LGA at birth was found to be a significant predictor of weight at 2 year.

### 3.2. Evidence in Pregnancy Complicated by Gestational Diabetes Mellitus

Table 2 summarizes the characteristics of the three (two epidemiological and one interventional) studies in GDM pregnancies.

#### 3.2.1. Epidemiological Studies

In the Nurses' Health Study II [21], there were 758 cases of GDM among 13,110 eligible pregnant women. Prepregnancy GI and GL were assessed by a validated 133-item semiquantitative FFQ. Prepregnancy GL was the only dietary factor found to be positively related to the risk of developing GDM (multivariate adjusted relative risk (RR) of the highest quintile versus the lowest quintile = 1.61; 95% CI = 1.02–2.53; *P* for trend =.03). The risk was increased 2.2-fold (95% CI: 1.04–4.29) among women with the highest prepregnancy GL and lowest fibre intake. Women who had a prepregnancy dietary GI > 57 also had a significantly higher risk of developing GDM compared to those who had a prepregnancy dietary GI < 51.0 (multivariate adjusted RR = 1.30; 95% CI 1.00–1.68) though the trend was marginally nonsignificant (*P* = .07). However, a smaller-scale study by Radesky et al. [22], which assessed the prepregnancy GL by the same FFQ used by Zhang et al. [21] failed to find any association between prepregnancy GL and risk of developing GDM.

#### 3.2.2. Intervention Studies

Direct evidence to support the use of a low-GI diet during pregnancy complicated by GDM is currently limited, with only one such study found in the literature search. Moses et al. [23] found that a significantly higher proportion of women in the higher GI group met the criteria to commence insulin than women in the low GI group (59% versus 29%; *P* = .023). In addition, nine out of 19 women in the high GI group who met the criteria for insulin commencement avoided insulin by switching to a low-GI diet. No significant differences in key fetal and obstetric outcomes were found.

## 4. Discussion

Among the eight studies investigated in this systematic review, four showed a protective association between low GI/GL and pregnancy-related outcomes, three showed no association, while one showed a potential increase in SGA risk. More studies are required to provide a convincing evidence base to support/reject the routine use of a low-GI diet in pregnancy. The current evidence suggests that the risk associated with a low-GI diet during pregnancy is minimal.

Traditionally, pregnancy diets recommended by health groups [24, 25] and government authorities [26–28] focus on nutrient adequacy because the requirements for many nutrients increase during pregnancy [29]. These recommendations, however, do not acknowledge any specific consideration of the glycemic potency of the foods in the diet. Many common staples such as rice, white bread, and potatoes, while nutritious, are high GI. The typical pregnancy diet is therefore of moderate to high GI [19, 23], depending on carbohydrate distribution and proportions of high-GI starchy foods versus low-GI foods such as fruit and dairy products. 

Elevated maternal blood glucose levels are well recognised as contributing to excessive fetal growth [13]. Among women with unrecognized maternal gestational diabetes mellitus (GDM), the prevalence of LGA infants is fivefold higher compared to nondiabetic controls and twofold higher compared to diet-controlled GDM women [30]. The HAPO study also provided robust evidence that maternal hyperglycemia 1-hour after a 75 g oral glucose tolerance test (OGTT), even within the recommended ranges, increases adverse pregnancy outcomes [31], and the risks increased further as the 1-hour postload blood glucose level rose. The 75 g OGTT can be regarded as a surrogate marker of meal postprandial glycemia. Therefore, maternal hyperglycemia (fasting, after a glucose load, and possibly postprandial) is likely to lead to adverse pregnancy outcomes [32].

Interventions that reduce maternal postprandial blood glucose levels, including dietary strategies, have been found to be effective in reducing macrosomia (birth weight > 4 kg) and childhood obesity in diabetic pregnancies [12, 19]. Moderate carbohydrate restriction is the most straight forward and commonly used strategy to achieve this as carbohydrates are the main determinant of postprandial blood glucose level [33]. However, a recent meta-analysis of randomized clinical trials among normal pregnant women showed lack of benefits of increasing protein intake in place of carbohydrate and the potential for increased risk for small-for-gestational-age (SGA) babies [34]. For this reason, reduction of maternal postprandial glycaemia by substituting dietary carbohydrate with protein may not be recommended in healthy pregnancies at the present time.

On the other hand, postprandial glycemia can be reduced without carbohydrate restriction by slowing down the rate of carbohydrate digestion and absorption. Compared to moderate- or high-GI foods containing similar amount of carbohydrates, low-GI foods have been demonstrated to reduce postprandial spikes of blood glucose level in healthy individuals [35]. A low-GI meal pattern therefore represents an alternative strategy for reducing postprandial glycemia in normal pregnancy without reducing the carbohydrate intake. The use of low-GI diets in normal pregnancy is controversial because any reduction in the rate of LGA may be matched by an increase in SGA, as has been shown in the epidemiological study by Scholl et al. [16] which reported an alarming increase of 75% in SGA risk. However, the rationale for assignment of GI values in their food database was not described and may not have been accurate. Women in the lowest quintile of GI also ate more refined sugar. Hence, poor overall dietary intake in this low-income population may have contributed to a contradictory finding and limits generalisation. Indeed the two intervention studies (one in normal pregnancy and one in GDM pregnancy) by Moses et al. [19, 23] showed that there is no significant increase in SGA in subjects following a low-GI diet. 

Because some low-GI foods have been associated with higher satiety [36, 37], a low-GI diet may also benefit pregnant women by reducing excessive maternal weight gain. High maternal weight gain has been linked to an increased risk of pregnancy complications [38], excessive fetal growth [39, 40], and long-term adverse health outcomes for the mother-infant pair [41]. The study by Deierlein et al. [17], however, reported no association between GL and total gestational weight gain, but total carbohydrate intake and GI were not reported separately. It is possible that a high intake of high-GI carbohydrates has a detrimental effect while a high intake of low-GI carbohydrate may be neutral or protective, as demonstrated in recent studies on risk of cardiovascular disease [42, 43]. 

Intervention studies in normal pregnancy are more supportive. The study by Clapp [18] was the first of its kind to investigate the effect of a low-GI diet on various pregnancy outcomes. While he reported results that favored the use of a low-GI diet during normal pregnancy, this study should be carefully interpreted. The number of subjects was small (6 in each group), the GI of the diets was not determined and the macronutrient proportions were not given. Differences in the amount of carbohydrate would also potentially affect outcomes. Expressed as a proportion of total energy intake, total carbohydrate intake has previously been shown to be associated with several pregnancy outcomes such as LGA and macrosomia, at least in pregnancy complicated with GDM [44, 45]. The more recent study by Moses et al. [19] on the other hand, provided stronger evidence that a low-GI diet improves pregnancy outcomes which is consistent with the findings of Clapp [18]. Unfortunately, the pregnant women in the study by Moses et al. reverted back to their baseline diet within 2 years [20], suggesting that dietetic followup may benefit these women particularly if they plan to become pregnant again, as prepregnancy GI and GL has been linked to increased risk of developing GDM [21].

It is now generally accepted that treating even mild GDM results in marked improvement in pregnancy outcomes. This view is supported by the large-scale Australian Carbohydrate Intolerance Study in Pregnant Women (ACHOIS) study [46] in which women with mild GDM were either treated intensively or attended routine antenatal care for healthy pregnancies. Intensive treatment in mild GDM, compared to routine care, resulted in reduced risks of preeclampsia, perinatal morbidity (e.g., shoulder dystocia), as well as macrosomia. The recent Maternal-Fetal Medicine Unit (MFMU) Network study [47] provided similar evidence. Even though lowering the dietary GI was not a specific aim of the dietary intervention in the ACHOIS study, it is likely that the GI was lowered because many of the healthy foods routinely recommended in pregnancy, such as fruit and dairy foods, are low GI. The dietary intervention in the MFMU study, on the other hand, may have incorporated low-GI foods as it was based on the American Diabetes Association position statement on “*Nutrition Recommendation and Intervention for Diabetes*” [48], which explicitly recommends “…*low-glycemic index foods that are rich in fibre and other important nutrients are to be encouraged.*”

Postprandial glucose excursion has been associated with adverse pregnancy outcomes in women with GDM [49]. Moderation of carbohydrate intake is usually recommended as the main and first-line strategy to achieve postprandial euglycemia [50]. However, there is evidence to suggest that overrestriction of carbohydrate in pregnancy complicated by GDM may increase the risk of fetal macrosomia [45], and therefore consideration to the glycemic potency of the carbohydrates in the diet is also important. By consuming low-GI carbohydrates one may achieve an adequate carbohydrate intake with lower postprandial blood glucose levels. The study by Moses et al. [23] suggested that a low-GI diet in GDM pregnancy can effectively reduce the need for insulin for optimal blood glucose management.

Clearly there is a lack of research in this area despite growing interest from the medical and nutrition community. In 2008, Tieu et al. [51] conducted a systematic review of dietary strategies for the prevention of GDM. They found only two trials [19, 52] (*n* = 82 in total) comparing the effect of a low-GI versus high-GI diets on obstetric outcomes and concluded that the evidence to support the use of a low-GI diet during pregnancy was inadequate, mainly due to the small number and the high heterogeneity of the trials available. A larger, randomized controlled trial investigating the effect of a low-GI diet on outcomes in GDM pregnancy, such as birth weight *z*-score, infant ponderal index, so forth, is currently underway [53]. More studies, particularly those which intervene at an earlier stage of pregnancy, are warranted.

## 5. Future Directions and Conclusions

Based on the currently available evidence, the use of a low-GI diet during pregnancy would appear to have no disadvantages. There is also some evidence that for women in general, and for women with special problems such as GDM, a low-GI diet can offer some advantages. However until further larger-scale intervention trials, preferably randomized controlled trials, are completed, a low-GI diet should not replace the current pregnancy recommendations from government and health agencies. Further research regarding the optimal time to start a low-GI diet for maximum protection of adverse pregnancy outcomes is also required.

## Figures and Tables

**Table 1:** Characteristics and outcome measures of studies examining the association between glycemic index/glycemic load and pregnancy outcomes in healthy pregnancies.

Study	Study characteristics	Exposure variables	Outcome variables	Summary of findings
*Epidemiological studies*				

Scholl et al. [16] 2004	*n* = 1, 082 Age: ≤18 y to 32 y Dietary assessment: 24-hour recall at 20- and 28-week gestation	GI by quintiles Q1: <50 versus Q5: >60	Birth weight SGA/LGA births	Dietary GI in the lowest quintile was associated with a statistically significant reduction of 116 g in birth weight, while dietary GI in the highest quintile was associated with a nonsignificant increase in birth weight (50.0 g) after adjustment for potential confounders. Compared to subjects with a dietary GI in Q3, those with a dietary GI in the lowest quintile had a 75% increased risk of giving birth to an SGA infant. No significant association was found between GI (in quintiles) and risk of LGA.

Deierlein et al. [17] 2008	*n* = 1,231 Age: ≥16 y Dietary assessment: semiquantitative FFQ at 26–29 weeks	GL by quartiles Q1: <112 versus Q4: >175.	Total gestational weight gain (TGWG) and weight gain ratio (WGR)	*No association* between GL and TGWG/WGR was found.

*Intervention studies*				

Clapp [18] 1997	*n* = 12 Mean age: 34.5	Aboriginal carbohydrate (low glycemic; GI = 50) diet versus cafeteria carbohydrate (high glycemic; GI = 59) diet together with exercise	Placental growth Birth weight Neonatal anthropometrics Maternal weight gain	Women who followed the cafeteria diet had a larger placental weight at delivery (575 ± 52 g versus 396 ± 18 g; *P* < .001). These women also gave birth to larger infants (*P* < .01) and gained more weight during pregnancy (*P* < .01)

Moses et al. [19] 2006	*n* = 62 Age: 21–40 y 16–20 week gestation at baseline	Low GI diet (GI = 51) versus high GI (GI = 58) diet	Method of delivery Maternal weight gain Birth weight Birth centile Head circumference Ponderal index Prevalence of LGA/SGA	Women who followed low-GI diet gave birth to lighter infants (*P* = .051), had lower birth centile (*P* = .005), and had a lower prevalence of LGA (*P* = .01). Their infants also had a lower ponderal index (*P* = .03). There was a nonsignificant increase of SGA prevalence.

Moses et al. [20] 2007	*n* = 43 Followup of Moses et al. [19] 2006 Age of infant: 16–29 months	Same as Moses et al. [19] 2006	GI of current diet Infant size	No difference was found in current dietary GI between subjects who followed the low-GI diet and those who followed the high-GI diet during pregnancy. LGA was a significant predictor of current infant weight (*P* = .037)

FFQ: food frequency questionnaire; SGA: small for gestational age (≤10th birth weight percentile); LGA: large for gestational age (≥90th birth weight percentile).

**Table 2:** Characteristics and outcome measures of studies examining the association between glycemic index/glycemic load and pregnancy outcomes in pregnancies complicated by gestational diabetes mellitus.

Study	Study characteristics	Exposure variables	Outcome variables	Summary of findings
*Epidemiological studies*				

Zhang et al. [21] 2006	*n* = 13,110 Mean age: 31.5 y Dietary assessment: 133-item semiquantitative FFQ, capturing prepregnancy dietary pattern	GI in quintiles Q1: <51 versus Q5: >57 GL in quintiles Q1: <104 versus Q5: >138	Incidence of GDM (*n* = 758)	Subjects with dietary GI in the highest quintile had a 30% increased risk of developing GDM while those in the highest quintile of GL had a 61% increased risk. There was also a significant increase in risk for increasing dietary GL (*P* = .03) while that for dietary GI was nonsignificant (*P* = .07)

Radesky et al. [22] 2008	*n* = 1,733 91 incidences of GDM Mean age: 31.5 y Dietary assessment: 133-item semiquantitative FFQ, at 5–25.6 weeks to capture prepregnancy dietary pattern	Per 22 units increase of GL	Incidence of GDM (*n* = 91)	*No association* between GDM risk and prepregnancy GL

*Intervention study*				

Moses et al. [23] 2009	*n* = 63 Mean age: 31.0 y Mean gestation weeks at baseline: 30.1 weeks	Low-GI diet (GI = 48) versus high-GI diet (GI = 56)	Need for insulin	Higher proportion (59% versus 29%; *P* = .023) of women following the high-GI diet required insulin for optimal GDM management. Switching from high-GI to low-GI diet helped 47.4% of these women avoid insulin. No significant differences in key fetal and obstetric outcomes were found.

GDM: gestational diabetes mellitus; FFQ: food frequency questionnaire.

## Appendix

### Search Strategy

1. Exp Glycemic Index/
2. glyc?emic index.tw
3. glyc?emic load.tw
4. exp Pregnancy Outcomes/
5. exp Diabetes, Gestational/
6. ((1) or (2) or (3)) AND ((4) or (5))
7. Limit 6 to (humans and yr=“1980–2010”).

This search strategy was used for MEDLINE and was slightly adapted for use with EMBASE, SCOPUS, CINAHL, and ISI Web of Science. We only included articles written in English.
